# Fibromatosis-like metaplastic carcinoma: a case report and review of the literature

**DOI:** 10.1186/s13000-020-00943-x

**Published:** 2020-03-03

**Authors:** Jasper Victoor, Claire Bourgain, Sara Vander Borght, Isabelle Vanden Bempt, Carine De Rop, Giuseppe Floris

**Affiliations:** 1 Department of Pathology, University Hospitals Leuven, Herestraat 49, 3000 Leuven, Belgium; 2 Department of Pathology, Imelda hospital, Bonheiden, Belgium; 3 Department of Human Genetics, University Hospitals Leuven, Leuven, Belgium; 4 Department of Gynecology, Imelda hospital, Bonheiden, Belgium; 5 Laboratory of Translational Cell & Tissue Research, Department of Imaging and Pathology, KU Leuven – University of Leuven, Leuven, Belgium

**Keywords:** Low-grade fibromatosis-like metaplastic carcinoma, Metaplastic breast carcinoma, Spindle cell lesion, Breast

## Abstract

**Background:**

We report an unusual case of low-grade fibromatosis-like metaplastic carcinoma (LG-FLMC) of the breast. This exceedingly rare epithelial breast malignancy has been reported only 68 times in the past 20 years, and is classified as a subtype of metaplastic breast carcinoma (MBC). It is a locally aggressive tumor with a low potential for lymph node and distant metastases, but with a tendency to recur after excision. Here we describe a less common presentation of LG-FLMC, provide its molecular characterization, discuss the major differential diagnosis and bring a short review of the literature.

**Case presentation:**

A 65-year-old woman presented with a self-palpated breast lump that had discordant radio-pathological features. While imaging results were compatible with an infiltrative malignancy, on core needle biopsy (CNB) a sharply delineated lesion composed by a bland-looking population of spindle cells was observed; excision was recommended for final diagnosis. Histology of the resection specimen showed small areas of epithelial differentiation and foci of peripheral invasion. Immunohistochemical analysis revealed a co-immunoreactivity for epithelial and myoepithelial markers in the spindle cell component. Mutation analysis with a capture-based next generation sequencing method revealed pathogenic mutations in *GNAS*, *TERT*-promotor and *PIK3R1* genes. A diagnosis of LG-FLMC was rendered.

**Conclusion:**

This case highlights the importance of a broad differential diagnosis, exhaustive sampling and the use of a broad immunohistochemical panel whenever dealing with a low-grade spindle cell lesion in the breast, and provides further insights into the molecular background of LG-FLMC.

## Introduction

Spindle cell lesions of the breast are infrequently encountered pathological entities, originating from a variety of cell types. They cover a wide spectrum including reactive processes, benign lesions and low- to high-grade malignancies [1–3]. Overlapping histological features are not uncommonly encountered in this type of lesion, making the diagnostic process a real challenge in some cases. It is, however, important to accurately recognize the pathology of these lesions, to avoid inappropriate management. Knowledge of the different morphological, immunohistochemical and molecular features and correlation with the clinicoradiological features are essential to eventually make the correct diagnosis [1, 4].

Occasionally, breast carcinomas may lose their epithelial morphology and show a pure spindle cell morphology, suggesting a transition towards epithelial-to-mesenchymal differentiation. Depending on nuclear atypia and mitotic activity, low-grade spindle cell carcinomas can be distinguished from the high-grade ones. Spindle cell carcinomas belong to the category of metaplastic breast carcinomas (MBCs), a broader and more heterogeneous group of malignant tumors [5].

Low-grade fibromatosis-like metaplastic carcinoma (LG-FLMC) represents a rare subtype of these spindle cell carcinomas of the breast, making up for < 1% of all invasive breast cancers [5, 6]. It is distinguished from other types of MBC because its unique resemblance to desmoid fibromatosis (DF), its tendency for local recurrence and its favorable prognosis [4, 6, 7, 9]. They have a low potential for lymph node or distant metastasis [4, 7]. Complete excision with adequate margins is therefore regarded as a curative treatment [4, 7].

Despite resembling a histologically benign mesenchymal-looking tumor, LG-FLMC is an epithelial malignancy that should be recognized and treated accordingly. Although its different morphological and immunohistochemical characteristics are well known in the literature, its molecular profile is poorly characterized to date. Here we report a case of LG-FLMC with unusual presentation and provide further insights into its molecular background.

## Case presentation

A 65-year-old woman presented with a self-palpated small lump in the left breast. She already underwent three breast lumpectomies in another hospital for benign lesions; two of the left breast and one of the right breast. She did not take any medications and had no family history.

Clinical examination confirmed the presence of a small nodule in the left breast, situated medially at 9 o’clock. Mammography and echography revealed a spherical, homogenous, non-cystic, well-defined mass of 19 × 14 × 19 mm. The lesion was situated close to the pectoralis major muscle and was suspicious for muscle invasion. Doppler ultrasonography revealed an important perilesional and peripheral vascularization. Several enlarged ipsilateral axillary lymph nodes were noticed.

**Fig. 1:**
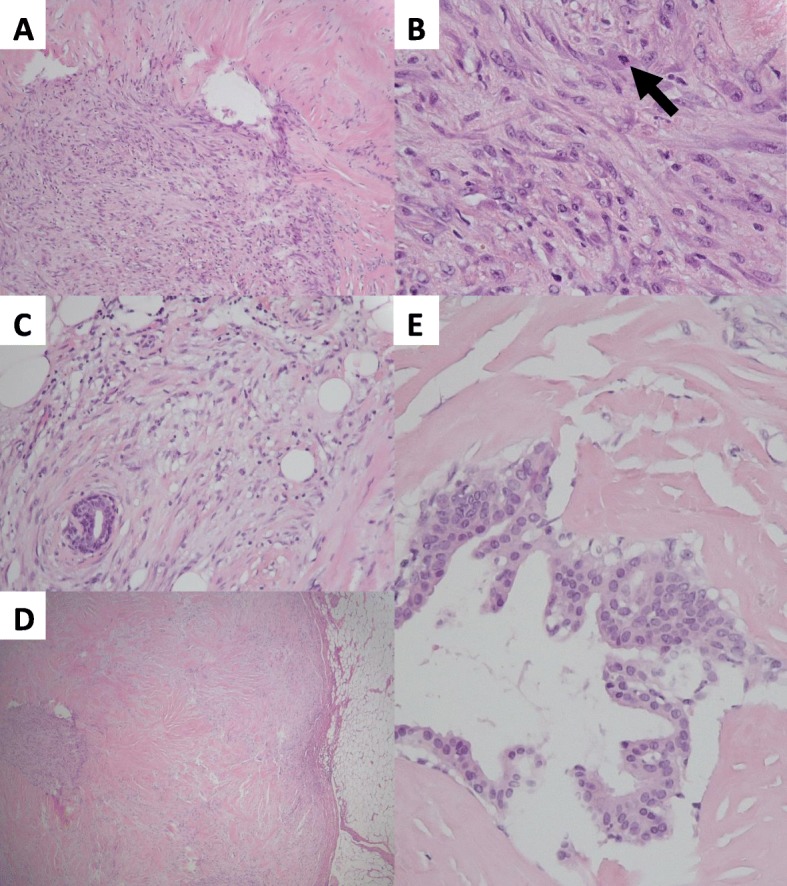
Routine histology with hematoxylin and eosin (H&E) stains. **a** The breast lesion has a variable cellularity. Large areas consist of dense collagenous stroma with a low cellularity (upper right of the picture). These areas are admixed with more cellular areas that have a myxoid appearance (lower left of the picture). (H&E ×50). **b** A highly cellular, myxoid area of the lesion. There is clustering of fusiform to discrete epithelioid cells with a round, slightly irregular nucleus that contains a small nucleolus. Occasional mitotic figures are seen (arrow). (H&E × 400). **c** Within the breast lesion, small and normal-looking ducts are entrapped by the proliferative spindle cells. The latter show no obvious atypia or mitotic activity at high magnification. At the periphery, a limited infiltration of bland-looking spindle cells into the surrounding fat tissue is seen. A scattered infiltrate of lymphocytes is also seen. (H&E × 100). **d** A view of the assessable border of the breast lesion at low magnification. A nodular and sharply delineated margin in relation to the surrounding fat tissue can be seen. This is not typical for FLMC, where a more infiltrative growth pattern is expected at the border. (H&E × 25). **e** In the center of the lesion, a distinctive area with a relatively high number of bland-looking ducts surrounded by a striking sclerotic stroma is seen. (H&E × 200)

Core needle biopsies (CNBs) were taken from one enlarged axillary lymph node and from the breast lump. The latter showed a spindle cell lesion with a sharply defined round border that separated the lesion from the surrounding fat tissue. At higher magnification, a variable cellularity was observed but no infiltrative permeation in the surrounding tissue was noticed. Both poorly and highly cellular areas consisted of bland-looking spindle cells. While the poorly cellular areas were characterized by a dense compact collagenous stroma, the more crowded areas showed haphazardly arranged cells embedded in loose connective tissue with myxoid appearance (Fig. 1a). Mitotic figures were absent. The CNB taken from the lymph node showed no malignancy.

Because of the discrepancy between the radiological findings (suggestive for an infiltrative malignancy) and the pathological findings (consistent with a bland-looking, sharply delineated spindle cell lesion), a complete local resection was recommended for definitive diagnosis.

At gross examination, the excised lesion presented as a sharply delineated nodule with homogenous white color, hard consistency and regular borders. The nodule had a diameter of 19 mm and was completely resected with a free margin of 8 mm. The lesion was surrounded by adipose tissue; no muscular tissue was resected during the surgical procedure.

In addition to the morphological features observed in the CNB, we noticed the presence of small clusters of epithelioid cells admixed with the spindle cells in the highly cellular fields of the resection specimen. The epithelioid cells contained oval nuclei with vesicular aspect, slightly irregular borders and often a prominent nucleolus. Occasional mitotic figures were present, as opposed to the CNB (Fig. 1b). Focally, despite a mainly sharply delineated margin, we also noticed small areas of invasion in the surrounding adipose tissue at the periphery of the lesion (Fig. 1c, d). In the areas of peripheral invasion, several entrapped ducts could be found, often surrounded by scattered lymphocytes. A distinctive central scar-like sclerotic zone admixed with bland-looking ducts was noticed as well (Fig. 1e). We considered the central scar-like sclerotic zone as a pre-existing sclerotic lesion. The spindle cell lesion was completely resected, albeit with a minimal margin of less than 1 mm.

Based on these morphological features, we considered a broad differential diagnosis including DF, adenomyoepithelioma (AME), inflammatory myofibroblastic tumor (IMT), myofibroblastoma, pseudoangiomatous stromal hyperplasia (PASH) and LG-FLMC. A broad panel of immunohistochemical stains was performed to narrow this differential diagnosis.

**Fig. 2:**
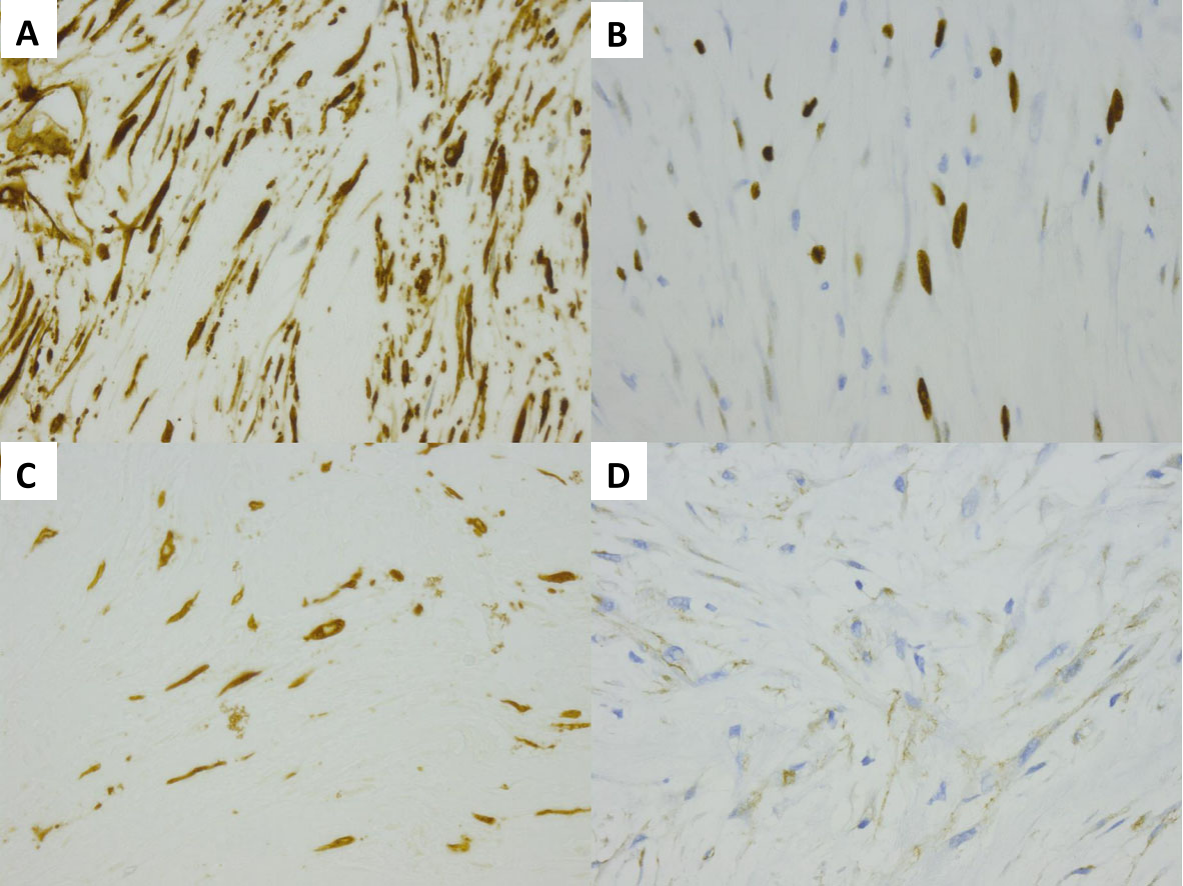
Immunohistochemistry. Staining patterns for αSMA (**a**), p63 (**b**), CK AE1/AE3 (**c**) and β-catenin (**d**). (IHC × 400). There is a co-expression in both the spindle cells and the more epithelioid cells for p63 and CK AE1/AE3

The spindle cells showed a diffuse immunoreactivity for alpha smooth muscle actin (α-SMA) (Fig. 2a), p63 (Fig. 2b), and cytokeratin AE1/AE3 (Fig. 2c). Focal immunoreactivity for S100, desmin and caldesmon was also noticed. There was no immunoreactivity for estrogen receptor (ER), progesterone receptor (PR), human epidermal growth factor receptor 2 (HER2), CD34, B-cell lymphoma 2 (Bcl-2), CD10 and anaplastic lymphoma kinase (ALK). β-catenin immunostaining showed focal cytoplasmatic staining, but no nuclear expression (Fig. 2d). The Ki67 immunostaining revealed a labeling index of about 2%, on average.

Because of the lack of expression for ALK, CD34 and ER and because of the lack of nuclear β-catenin expression, we excluded the diagnosis of IMT, myofibroblastoma, PASH and DF. Given the spindle cell morphology, the presence of rare epithelioid cells, the mitotic activity, the clear co-immunoreactivity for CK AE1/AE3 and p63 and the triple-negativity for ER, PR and HER2, the differential diagnosis was limited to a borderline malignant ER-negative AME with myoepithelial overgrowth and LG-FLMC. Notably, a sharply delineated border and association with a centrally sclerotic region may be observed both in AME and LG-FLMC [3, 10].

The further characterization of this lesion included mutational analysis of 97 cancer related genes (Table S1) with capture-based targeted next generation sequencing method in search of recurrent mutations in the *HRAS* and/or *PIK3CA* gene that could have helped in the differential diagnosis [3, 11–13]. The following pathogenic variants were detected: *GNAS* c.601C > T p.(Arg201Cys) with variant allele frequency (VAF) 22%, *TERT*-promotor c.-124C > T p.? with VAF 20%, *PIK3R1* c.1365_1367del p.(Gln455_Phe456delinsHis) with VAF 21% and *PIK3R1* c.2088dup p.(His697Thrfs*44) with VAF 22%. No mutations were found in *HRAS* and *PIK3CA*.

Eventually we favored the diagnosis of LG-FLMC based on the morphological and immunohistochemical findings.

Because of the negative resection margins (albeit with a margin less than 1 mm), the favorable prognosis of this entity and the lack of proof of usefulness, no adjuvant therapy was given. After 2 years of clinicoradiological follow-up, the patient was still free of disease.

## Discussion

Spindle cell lesions of the breast can be morphologically subdivided roughly into high-grade lesions with malignant appearance and low-grade lesions with bland-looking aspect [1]. While in the former group the most important issue is to distinguish high-grade spindle cell MBC from rarer primary and metastatic malignancies like malignant phyllodes, sarcomas and melanoma [1]; in the latter group the most important issue is to distinguish benign lesions like DF, myofibroblastoma or nodular fasciitis from rarer low-grade malignancies like FLMC [1]. Overlapping morphological features are not uncommon among these entities, therefore a broad panel of immunostaining is recommended to solve the differential diagnosis. as illustrated by our case.

Various low-grade spindle cell lesions of the breast may enter in the differential diagnosis with FLMC including nodular fasciitis, (exuberant) scar tissue, myofibroblastoma, IMT, PASH, solitary fibrous tumor, phyllodes tumor, dermatofibrosarcoma protuberans, melanoma and primary angiosarcoma [1,7,9]. Combination of morphology, clinical history, imaging, immunohistochemistry and molecular pathology can help further in establishing the diagnosis. In this regard, our initial differential diagnosis included also myofibroblastoma, IMT and PASH. The negative expression of ALK, ER and CD34 together with the morphology helped us to exclude these entities. However, DF and AME with overgrowth of the spindle cell component remained in our opinion the two major entities in the differential diagnosis with FLMC. We will further focus especially on these latter entities in our discussion.

Our case presented as a predominantly nodular lesion with only a focally infiltrative growth pattern that was apparent only in the resection specimen. Interestingly, our case was in relation with a centrally located pre-existing sclerosing lesion. At first sight, many morphological features are shared by both DF and LG-FLMC, including the growth pattern with finger-like projections invading the surrounding mammary tissue and the bland looking spindle cells embedded in collagenous stroma with variable texture [1, 7, 9]. However, FLMC may present as exclusively nodular or with pushing margins with focal invasion, like in our case [7, 9]. Despite the fact that in FLMC over 90–95% of the tumor cell population is represented by fibroblast-like spindle cells with mild to no atypia, clusters of epithelioid cells or less frequently glandular and squamous epithelial elements can be focally present in FLMC [1, 9]. This detail represents a useful clue to address the differential diagnosis. Moreover, when dealing with larger lesions a thorough sampling of the tumor is recommended to avoid missing small area of epithelioid differentiation. In this regard, the CNB in our case illustrates the risks of undersampling in this type of lesions. By immunohistochemistry, epithelial origin should be demonstrated preferentially by a broad-spectrum cytokeratin stains which includes both low and high molecular weight cytokeratins like CK AE1/AE3, MNF116, CK5,6, CK14 and 34βE12 [7, 9]. The expression of these epithelial markers in the spindle cell component of the tumor is the signature of MBC [4, 9], however the choice for a broad panel of antibodies is justified by the fact that MBC with spindle cell morphology can occasionally show only focal cytokeratin expression [1]. Additionally, the co-expression of the myoepithelial marker p63 in the spindle cell component is proven to be a sensitive and specific diagnostic feature [9] which should be also included in the panel of immunostainings. Other myoepithelial markers, such as CD10, calponin and α-SMA may be also expressed [4, 6]. DF does not show expression for any of the above-mentioned markers except for α-SMA, and is typically characterized by nuclear expression of the β-catenin protein which generally reflects in over 80% of the cases the presence of an underlying mutation in the *CTNNB1* gene [1, 9]. Recent evidence suggest that DF of the breast, as compared to DF of other sites, may have a lower frequency of *CTNNB1* and higher rate of *APC* gene mutations [14]. Rarely, spindle cell MBCs can show nuclear immunoreactivity for β-catenin as well, but in these cases the staining pattern is usually focal and weak [1, 15]. Interestingly the lymphoid enhancer binding factor 1 (LEF1), which is part of the Wnt signaling pathway together with β-catenin, has been recently proposed as sensitive and specific marker for DF in certain context [16].

Although not typically mentioned in the differential diagnosis of spindle cell lesions of the breast, a borderline malignant AME can be a potential differential diagnosis when it comes to FLMC. Morphologically both entities may show nodular aspect, may be associated to pre-existing sclerotic lesions and may show heterogeneous cellular composition [1, 3, 7, 9]. AME is mostly considered a benign tumor, often associated to papillary lesion and characterized by proliferation of both myoepithelial and glandular component. However, in some instances the myoepithelial component may be prevalent and show spindle cell morphology which can be the only assessable component in small samples. The presence of enhanced mitotic activity, mild nuclear atypia or invasive growth pattern in an otherwise benign looking AME all are considered worrisome morphological features that may justify the diagnosis of borderline malignant AME. To further underline the morphological similarities between AME and MBC with spindle cell morphology, it is important to mention that in malignant AMEs the malignant epithelial component is often of the metaplastic subtype while myoepithelial carcinoma was regarded as MBC in the past edition of the WHO classification of breast tumors [*WHO classification of breast tumours.* 5th ed. Houston, IARC; 2019, in press]. Both AME and FLMC show broad immunohistochemical overlap, however the pattern of staining of the CK AE1/AE3 marker might be helpful in the differential diagnosis because in AME, as opposed to FLMC, only the epithelial cells and not the (spindled) myoepithelial cells show expression with this marker [9, 10]. FLMC and the majority of MBCs usually do not express ER, PR, HER2 [1, 9], while AMEs can be ER-positive.

From a molecular point of view, mutations in *PIK3CA*, *PIK3R1* and *PTEN* genes are significantly more frequently found in MBC as compared to triple-negative breast carcinomas of no special type [17]. *TERT*-promotor mutations are relatively more frequently associated to MBC with spindle cell morphology as compared to other types of MBCs [18, 19]. They are, however, frequently mutated in several other types of cancers and represent one of the most frequently observed mutations after *TP53* gene mutation. Interestingly, *TP53* mutations seem to be less common in spindle cell MBCs or other low-grade MBCs compared to matrix-producing or high-grade MBC subtypes [19]. This is well in line with our case, as we found two *PIK3R1* and a *TERT*-promotor mutation but no *TP53* mutations. Myoepithelial-like MBCs, as is FLMC, show frequently *CDKN2A* losses and recurrent mutations in the *PIK3CA* gene [13]. FLMCs in particular show low levels of genetic instability, with recurrent losses of *CDKN2A* and lack of recurrent mutations in *TP53*, *EGFR* and *KRAS* genes [3, 12]. In our case, losses of *CDKN2A* or mutations in *PIK3CA* could not be found. In contrast to FLMC, ER-negative AMEs harbor *HRAS*^*Q61*^ hotspot mutations that co-occur with *PIK3CA* or *PIK3R1* mutations in the majority of the cases [11]. Notably, *HRAS*^*Q61*^ mutations are clonal and seem to occur quite early in the pathogenesis of ER-negative AME, while *PIK3CA* or *PIK3R1* mutations seem to be only subclonal and may suggest a later acquisition in time, together with other genetic changes like *TERT*-promoter mutations and homozygous deletions of the *CDKN2A* gene [11]. By targeted massively parallel sequencing analysis we found in our case two *PIK3R1* mutations, but because of the absence of *HRAS*^*Q61*^ mutations together with morphology and immunohistochemistry, we excluded the diagnosis of borderline malignant ER-negative AME and we favored the diagnosis of an FLMC. To our knowledge, we are the first to report a mutation in the *GNAS* gene for FLMC in particular. Somatic *GNAS* mutations are frequently encountered in pituitary adenomas and in patients with intraductal papillary mucinous neoplasm of the pancreas. Germline *GNAS* mutations are associated with McCune-Albright syndrome and fibrous dysplasia. Notably, Bataillon et al. [20] also recently reported a *GNAS* mutation in low-grade adenosquamous carcinoma, which is another type of low-grade MBC. However, its significance in the pathogenesis of LG-FLMC is unclear and confirmation in a larger cohort is warranted.

FLMC is mostly encountered in postmenopausal women who typically present with a rapidly growing and palpable lump. There is no predilection for a specific side [9]. The radiological appearance is variable, ranging from benign-looking to highly suspicious for malignancy [4, 9]. Macroscopically, FLMC is a non-encapsulated firm white mass that has been described as nodular to irregular, sharply delineated to infiltrative and even cystic [4, 6, 7, 9]. Calcification, hemorrhage and necrosis are unusual findings [7–9]. Regarding prognosis, FLMC has a clinically indolent behavior with a high tendency for local recurrence but with low potential for lymph node or distant metastasis [4, 7–9]. Complete excision with adequate margins is therefore regarded as a curative treatment [4, 7]. However, distant metastases are still possible. Indeed, of the 68 cases of FLMC cases that we have found in the English literature of the past 20 years, 4 cases had distant metastases; rendering the use of the term ‘carcinoma’ appropriate (Table 1) [4, 8, 17]. The review of the English literature suggests that metastatic disease in FLMC seems to be related to larger size of the primary tumor, while the risk of local recurrence seems to be related to inadequate local resection. Therefore, resection with wide margins is strongly recommended. Because of the low potential for lymph node metastasis, axillary lymph node dissection is not advised [7, 9]. Whether adjuvant radiotherapy or chemotherapy treatment could be useful to lower the risk of local recurrence or metastasis is not yet proven, but some authors argue for the use of adjuvant radiotherapy in voluminous lesions [4].

**Table 1:** Case reports of FLMC in English literature

Variable	Number of cases	Mean age (Range), years	Mean tumor size (Range), cm	Number of lymph node metastases	Number of distant metastases	Initial treatment	Adjuvant therapy	Molecular analysis	Recurrence (interval range, months)	Follow-up time, months
**Nonnis et al. 2012** [4]	1	73	2.0	0	0	LE	No	NA	1 (9)	73; Second recurrence: 2 months after reexcision, treated by WE
**Zhao et al. 2018** [6]	3	57 (51–65)	3.5 (3.0–4.0)	0	0	2 MRM 1 WE	2 CT 1 CT + RT	NA	1 (13)	Range 12–49
**Gobbi et al. 1999** [7]	30	63 (40–80)	2.7 (1.2–7.0)	0	0	5 MRM 5 WE+LN 5 WE 8 LE 7 NA	1 RT 1 CT 1 CT + RT 1 RT + HT	NA	8 (5–72); 7 after LE 1 after WE 0 after AT	Range 6–88 (18/30 cases); 1 with second recurrence 9 months after reexcision, treated by MRM
**Sneige et al. 2001** [8]	24	66 (55–85)	2.8 (1.0–5.0)	0	2; 1 lung 1 lung, inguinal soft tissue and bone; DOD 17–19 mos.	12 MRM 1 MRM with nCT 1 LE + LN 6 LE4 NA	5 RT 1 CT	NA	2 (5–32); 2 after LE 0 after AT	Range 5–90 (18/24 cases); No second recurrence
**Kinkor et al. 2002** [21]	4	(54–72)	(2.0–3.5)	NA	2; DOD	NA	NA	NA	NA	NA
**Schafernak et al. 2006** [22]	1	59	3.0	NA	NA	LE	NA	NA	NA	NA
**Rekhi et al. 2007** [23]	1	77	2.0	0	0	LE	RT	NA	NR	16
**Podetta et al. 2009** [24]	2	79 (72–85)	4.4 (3.0–5.7)	0	0	1 MRM 1 WE+LN	1 RT	NA	NR	Range 21–27
**Pagnon et al. 2017** [25]	1	66	NA	0	0	LE	RT	NA	NA	NA
**Victoor et al. 2020**	1	65	1.9	0	0	LE	No	Mutations in - *GNAS* - *TERT*-promotor - *PIK3R1* (×2)	NR	12

*FLMC* fibromatosis-like metaplastic carcinoma of the breast, *NA* not available, *DOD* died of disease, *LE* lumpectomy, *MRM* modified radical mastectomy, *WE* wide excision, *LN* axillary lymph node dissection, *(n)CT* (neoadjuvant) chemotherapy, *RT* radiotherapy, *HT* hormonal therapy, *NGS* next generation sequencing, *AT* adjuvant therapy

## Conclusion

Low-grade fibromatosis-like metaplastic carcinoma of the breast is a rare low-grade subtype of metaplastic breast carcinoma with a broad differential diagnosis. A wide panel of immunohistochemical stains should be taken under consideration when dealing with small biopsies, in order to assess a correct diagnosis. Exhaustive sampling of the resection specimen is recommended to avoid missing focal areas of epithelial differentiation, which is an important clue for the final diagnosis. Despite FLMC not being associated with specific recurrent mutations, *PIK3R1, PTEN* and *TERT*-promotor mutations are not uncommonly associated with MBCs with spindle cell morphology (including FLMC). FLMCs are locally aggressive and have an increased risk of local recurrence, with distant metastases only occasionally reported in the literature.

## Supplementary information

**Additional file 1: Table S1.** List of the 97 cancer related genes that were analyzed with capture-based targeted next generation sequencing method.

## Data Availability

A list of the 97 cancer related genes that were analyzed with capture-based targeted next generation sequencing method is available in a supplementary information file (Table S1). The raw data of the 97 genes that were analyzed with a capture-based targeted next generation sequencing method for this particular case is available at the Department of Human Genetics, University Hospitals Leuven, Leuven, Belgium on reasonable request.

## Abbreviations

ALK: Anaplastic lymphoma kinase
AME: Adenomyoepithelioma
Bcl-2: B-cell lymphoma 2
CK: Cytokeratin
CNB: Core needle biopsy
DF: Desmoid fibromatosis
ER: Estrogen receptor
HER2: Human epidermal growth factor receptor 2
IMT: Inflammatory myofibroblastic tumor
LEF1: Lymphoid enhancer binding factor 1
LG-FLMC: Low-grade fibromatosis-like metaplastic carcinoma of the breast
MBC: Metaplastic breast carcinoma
PASH: Pseudoangiomatous stromal hyperplasia
PR: Progesterone receptor
VAF: Variant allele frequency
α-SMA: alpha smooth muscle actin
