# Supplementary material for: Fibromatosis-like metaplastic carcinoma: a case report and review of the literature
Source: Diagn Pathol. 2020 Mar 3;15:20. doi: 10.1186/s13000-020-00943-x (PMC7053053; doi:10.1186/s13000-020-00943-x)
Supplement: Supplementary file 1 — Additional file 1: Table S1. List of the 97 cancer related genes that were analyzed with capture-based targeted next generation sequencing method. [file 13000_2020_943_MOESM1_ESM.docx]

**Supplemental Information**

Linked to the online version of the paper entitled: ***“Fibromatosis-like metaplastic carcinoma: a case report and review of the literature”***, by Victoor J and co-workers at Diagnostic Pathology / BioMed Central.

**Table S1: List of the 97 cancer related genes that were analyzed with capture-based targeted next generation sequencing method.**

| ***AKT1*** | ***CTNNB1*** | ***GNA11*** | ***NF1*** | ***SDHA*** |
| --- | --- | --- | --- | --- |
| ***ALK*** | ***DDR2*** | ***GNAQ*** | ***NF2*** | ***SDHB*** |
| ***AMER1*** | ***DICER1*** | ***GNAS*** | ***NOTCH1*** | ***SDHC*** |
| ***APC*** | ***DPYD*** | ***H3F3A*** | ***NRAS*** | ***SDHD*** |
| ***ARAF*** | ***EGFR*** | ***H3F3B*** | ***PALB2*** | ***SMAD4*** |
| ***ARID1A*** | ***ERBB2*** | ***HIST1H3B*** | ***PDGFRA*** | ***SMARCA4*** |
| ***ATM*** | ***ERBB3*** | ***HIST1H3C*** | ***PDGFRB*** | ***SMARCB1*** |
| ***ATRX*** | ***ERBB4*** | ***HRAS*** | ***PIK3CA*** | ***SMO*** |
| ***BAP1*** | ***ERCC2*** | ***IDH1*** | ***PIK3R1*** | ***SPRED1*** |
| ***BCOR*** | ***ESR1*** | ***IDH2*** | ***POLE*** | ***STK11*** |
| ***BRAF*** | ***FBXW7*** | ***KIT*** | ***PRDM6*** | ***SUFU*** |
| ***BRCA1*** | ***FGFR1*** | ***KRAS*** | ***PTCH1*** | ***TERT*** |
| ***BRCA2*** | ***FGFR2*** | ***LZTR1*** | ***PTEN*** | ***TP53*** |
| ***CCND1*** | ***FGFR3*** | ***MAP2K1*** | ***RAC1*** | ***TSC1*** |
| ***CCNE1*** | ***FGFR4*** | ***MDM2*** | ***RAF1*** | ***TSC2*** |
| ***CDH1*** | ***FOXL2*** | ***MDM4*** | ***RB1*** | ***VEGFR3*** |
| ***CDK4*** | ***FUBP1*** | ***MET*** | ***RET*** | ***WT1*** |
| ***CDK6*** | ***GATA3*** | ***mTOR*** | ***RICTOR*** |  |
| ***CDKN2A*** | ***GLI1*** | ***MYC*** | ***RNF43*** |  |
| ***CIC*** | ***GLI2*** | ***MYCN*** | ***ROS1*** |  |

This table provides a list of the 97 genes that were analyzed with capture-based targeted next generation sequencing method. The genes are listed in alphabetical order.
